# MRI features of idiopathic intracranial hypertension are not prognostic of visual and headache outcome

**DOI:** 10.1186/s10194-023-01641-x

**Published:** 2023-07-28

**Authors:** Gabriel Bsteh, Wolfgang Marik, Nik Krajnc, Stefan Macher, Christoph Mitsch, Philip Pruckner, Klaus Novak, Christian Wöber, Berthold Pemp

**Affiliations:** 1 Department of Neurology, Medical University of Vienna, Vienna, Austria; 2 Comprehensive Center for Clinical Neurosciences & Mental Health, Medical University of Vienna, Vienna, Austria; 3 Department of Neuroradiology, Medical University of Vienna, Vienna, Austria; 4 Department of Ophthalmology, Medical University of Vienna, Vienna, Austria; 5 Department of Neurosurgery, Medical University of Vienna, Vienna, Austria

**Keywords:** Idiopathic intracranial hypertension, Magnetic resonance imaging, Visual outcome, Empty sella, Perioptic subarachnoid space distension, Optic nerve tortuosity, Posterior globe flattening, Transverse sinus stenosis

## Abstract

**Background:**

In idiopathic intracranial hypertension (IIH), certain MRI features are promising diagnostic markers, but whether these have prognostic value is currently unknown.

**Methods:**

We included patients from the Vienna-Idiopathic-Intracranial-Hypertension (VIIH) database with IIH according to Friedman criteria and cranial MRI performed at diagnosis. Presence of empty sella (ES), perioptic subarachnoid space distension (POSD) with or without optic nerve tortuosity (ONT), posterior globe flattening (PGF) and transverse sinus stenosis (TSS) was assessed and multivariable regression models regarding visual outcome (persistent visual impairment/visual worsening) and headache outcome (headache improvement/freedom of headache) were fitted.

**Results:**

We included 84 IIH patients (88.1% female, mean age 33.5 years, median body mass index 33.7). At baseline, visual impairment was present in 70.2% and headache in 84.5% (54.8% chronic). Persistent visual impairment occurred in 58.3%, visual worsening in 13.1%, headache improvement was achieved in 83.8%, freedom of headache in 26.2%.

At least one MRI feature was found in 78.6% and 60.0% had ≥3 features with POSD most frequent (64.3%) followed by TSS (60.0%), ONT (46.4%), ES (44.0%) and PGF (23.8%).

In multivariable models, there was no association of any single MRI feature or their number with visual impairment, visual worsening, headache improvement or freedom.

Visual impairment at baseline predicted persistent visual impairment (odds ratio 6.3, p<0.001), but not visual worsening. Chronic headache at baseline was significantly associated with lower likelihood of headache freedom (odds ratio 0.48, p=0.013), but not with headache improvement.

**Conclusions:**

MRI features of IIH are neither prognostic of visual nor headache outcome.

**Supplementary Information:**

The online version contains supplementary material available at 10.1186/s10194-023-01641-x.

## Introduction

Idiopathic intracranial hypertension (IIH), a syndrome of elevated intracranial pressure (ICP) with increasing prevalence but unclear etiology, bears not only the risk of visual impairment and chronic disabling headache, but also significant reduction of quality of life and considerable socioeconomic costs [1, 2].

Estimating prognosis at the time of diagnosis is currently very challenging as there is a large degree of interindividual heterogeneity in visual and headache outcome and a paucity of reliable outcome predictors [3, 4].

Neuroimaging is required for establishing IIH diagnosis in order to rule out secondary causes of elevated ICP [5]. There are also various MRI signs indicative of IIH such as empty sella (ES) sign, perioptic subarachnoid space distension (POSD) with or without optic nerve tortuosity (ONT), posterior globe flattening (PGF), and transverse sinus stenosis (TSS) [6]. A MRI displaying at least three out of these four signs is highly specific and moderately sensitive for elevated ICP, but absence of these findings does not rule out IIH and may depend upon rater experience [7, 8].

Apart from aiding in diagnosis, MRI features of IIH might also have prognostic value. However, currently available evidence in this regard is scarce, methodologically limited by small sample sizes, largely lacks adjustment for confounders, and thus, has delivered conflicting results [6, 9–13]. Furthermore, studies on prognostic impact of MRI in IIH have focused on visual outcome, largely leaving headache outcome aside.

Therefore, here we aimed to determine whether MRI features of IIH provide value for predicting visual and headache outcome in a large and well-characterized real-world cohort applying a thorough and pre-defined approach with multivariable analyses.

## Methods

### Patients and definitions

For this retrospective cohort study, we used the Vienna Idiopathic Intracranial Hypertension (VIIH) database, which is jointly established by the Departments of Neurology and Ophthalmology, serving as both primary and reference center mainly for Vienna and its geographical catchment area. By November 30th 2022, a cohort of 151 patients with IIH according to modified Friedman criteria had been included [5]. Details of the VIIH database are described elsewhere [14]. Briefly, standardized VIIH case reports include demographic data, disease specific parameters as well as documentation of diagnostic and therapeutic procedures. Data are collected retrospectively at first visit and prospectively whenever the patient returns for scheduled follow-up or unscheduled visits. Specialized neurologists and neuro-ophthalmologists performed all examinations. Headache history is assessed by a combination of history and a headache diary. Headache phenotype is classified according to ICHD-3 as either migraine-like, tension-type headache-like or unclassifiable [15]. All patients were treated according to best practice based on recommendation of weight loss, pharmacological treatment with acetazolamide, topiramate and/or furosemide, and invasive treatment options such as ventriculoperitoneal (VP) shunt in case of treatment refractory papilledema.

For the present study, we included all patients with definite IIH or IIH-WOP according to Friedman criteria for whom initial diagnostic MRI with images on file and a minimum follow-up of 12 months were available. Patients with probable IIH or suggested IIH-WOP according to Friedman criteria were excluded as well as any patients with secondary causes of intracranial hypertension [5]. We also excluded patients if (1) MRI had been performed more than 4 weeks before first diagnostic LP, or (2) LP was performed before MRI.

MRI scans were done on 1.5 or 3T MR scanners. MRI protocols differed in some detail but included at least T1 and T2w sequences in two different planes for excluding structural lesions and a venous non-contrast MR angiography or T1 post gadolinium sequence for excluding suspected sinus vein thrombosis. MRI images were independently reviewed by a senior neuroradiologist with extensive experience in IIH imaging (W.M.), who was blinded for clinical data. MRI features of IIH were defined as follows. ES sign was evaluated by assessing the degree of suprasellar herniation of CSF into the in the sagittal plane applying a cut-off of moderate herniation (≥ 1/3 of the sella height) [16]. POSD was defined as a uni- or bilateral optic nerve sheath width > 4 mm in the coronal plane of T2 weighted images [17]. ONT and PGF were based on a qualitative evaluation on axial T2 weighted images [17]. For TSS, the patency of each transverse sinus (left and right) was evaluated relative to the diameter of the lumen of the distal superior sagittal sinus with the narrowest segment of the transverse sinus used to determine the degree of stenosis. TSS was defined as uni- or bilateral stenosis of at least ≥ 50% [17, 18].

Headache and visual outcomes were assessed 12 months after IIH diagnosis applying the following definitions:

- Persistent visual impairment: visual acuity ≥ 0.1 logarithm of the minimum angle of resolution (logMAR; determined by Sloan charts at distance after subjective refraction) and/or mean deviation <-2.0 in decibels (dB) in automated static threshold perimetry determined by 30 − 2 test with Swedish interactive threshold algorithm (SITA) [19].
- Visual worsening: deterioration of visual acuity by ≥ 0.2 logMAR and/or mean deviation by ≥ 2.0 dB in static threshold perimetry compared to baseline.
- Headache improvement: ≥50% reduction of headache severity (on the visual analogue scale) and/or headache frequency (expressed as headache days per month) compared to baseline.
- Freedom of headache: <1 headache day per month.

As relevant covariables, visual impairment at baseline was defined as baseline visual acuity ≥ 0.1 logMAR and/or mean deviation <-2.0 dB in automated static threshold perimetry. Headache was classified as chronic if present on ≥ 15 days/month for ≥ 3 months.

### Standard protocol approvals, registrations, patient consents, and reporting

The study was approved by the ethics committee of the Medical University Vienna (ethical approval number: 2216/2020). As this was a retrospective study, the need for written informed consent from study participants was waived by the ethics committee. This study adheres to the reporting guidelines outlined within the Strengthening the Reporting of Observational Studies in Epidemiology (STROBE) Statement.

### Data availability statement

Data supporting the findings of this study are available from the corresponding author upon reasonable request by a qualified researcher and upon approval by the data-clearing committee of the Medical University of Vienna.

### Statistics

Statistical analysis was performed using SPSS 26.0 (SPSS Inc, Chicago, IL, USA) and R-Statistical Software (Version 4.0.0). Categorical variables were expressed in absolute frequencies and percentages, continuous parametric variables as mean and standard deviation (SD) and continuous non-parametric variables as median with inter-quartile range (IQR) and absolute range (AR) as appropriate.

Univariable group comparisons were done by Chi-squared test, Mann-Whitney U test or independent t-test (with Welch’s correction in case of unequal standard deviations between the groups) as appropriate. Univariable correlations were analyzed by Pearson or Spearman test as appropriate.

Association of MRI features with headache and visual outcome parameters was tested by Firth’s bias-reduced logistic regression models (R package “logistf”, Version 1.24.1), a penalized likelihood-based method which increases estimator efficiency in logistic regression models with small samples [20]. In these models, every single MRI sign was defined as a dichotomous independent variable comparing absence and presence of ES, POSD, ONT, PGF, and TSS using absence as reference category. Dependent variables comprised persistent visual impairment, visual worsening, headache improvement and freedom of headache. Similar models were set up for the presence and absence of a certain number of MRI signs, setting the cut-off at ≥ 1 and ≥ 3, respectively.

Corrected Akaike information criterion (AICc) was employed to select the most parsimonious models among a pre-defined set of covariables based on clinical gestalt (for visual outcomes: age, BMI, CSF opening pressure, visual impairment at baseline; for headache outcomes: age, BMI, CSF opening pressure, chronic headache at baseline, baseline headache severity) as well as any other variables available at baseline associated with outcome parameters at a p-value < 0.2 in univariable analyses [21].

Predefined sensitivity analyses to determine potential confounding influence were conducted with the same model set-up removing patients with a) IIH without papilledema (IIH-WOP), patients with previous headache history (only for models regarding headache outcomes), c) separately removing each headache phenotype group (migraine-like, tension-type-like, unclassifiable; only for models regarding headache outcomes) and d) patients requiring invasive treatment (VP shunt).

Robustness to unidentified confounders was quantified with Rosenbaum sensitivity test for Hodges–Lehmann Γ [22]. Missing values were handled by multiple (20 times) imputation using the missing not at random (MNAR) approach with pooling of estimates according to Rubin’s rules [23]. Significance level was set at a two-sided p-value < 0.05 with Bonferroni correction for multiple testing.

## Results

**Fig. 1:**
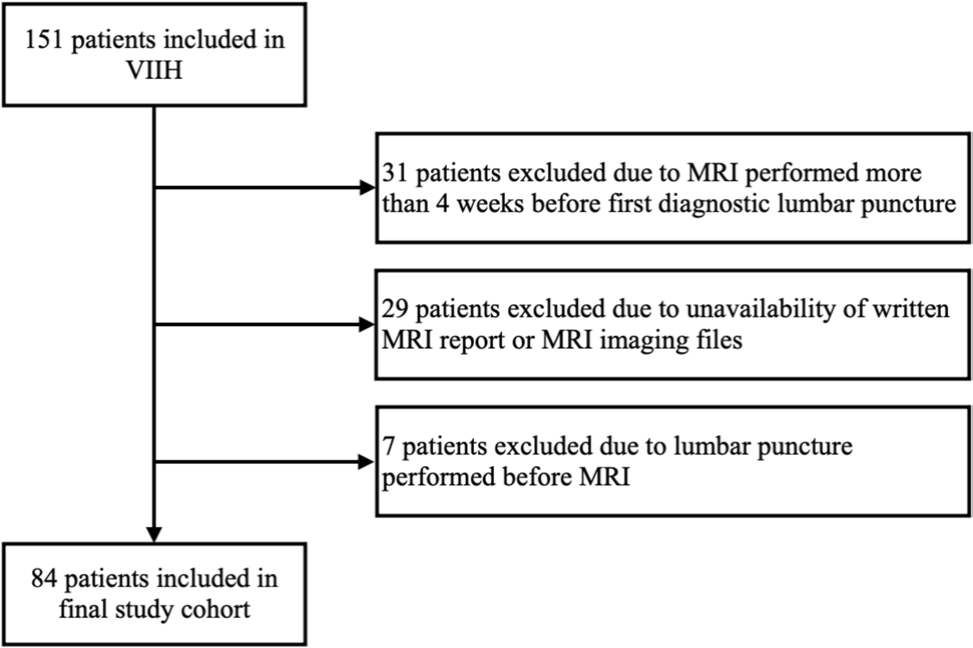
Flow chart of inclusion/exclusion process. MRI: magnetic resonance imaging. VIIH: Vienna Idiopathic Intracranial Hypertension database

Eighty-four patients were included into the final study cohort. The inclusion/exclusion process is shown in Fig. 1. Cohort characteristics at baseline are given in Table 1. Expectedly, there was a female predominance (88.1%) with mean age at diagnosis at 33.5 years (SD 11.3) and median BMI at 33.7 (IQR 27.3–39.0). Median LP opening pressure was 31 cmH2O (IQR 28–39). Five patients (6.0%) were diagnosed with IIH-WOP (3 fulfilling diagnostic criteria with unilateral abducens palsy, 2 with bilateral abducens palsy). Of note, cohort characteristics did not significantly differ from the whole VIIH cohort (data not shown).

Overall, ES sign was present in 44.4%, POSD in 64.3%, ONT in 46.4%, and PGF in 23.8%. Of the 70 patients with available venous MRI angiography, 60% displayed TSS. At least one MRI feature was found in 78.6% and ≥ 3 features in 60.0%. Neither any single MRI feature nor the number of MRI features was associated with age, sex, BMI, symptoms at initial presentation, ophthalmological findings or LP opening pressure.

**Table 1:** Cohort characteristics

	(*n* = 84)
Females^1^	74 (88.1)
Age at diagnosis^2^	33.5 (11.3)
Diagnosis	
IIH with papilledema^1^	79 (94.0)
IIH without papilledema^1^	5 (6.0)
BMI^3^	30.7 (27.3–39.0)
Overweight (BMI > 25)^1^	72 (85.7)
Previous headache history	33 (39.3)
Symptoms/signs at initial presentation	
Headache^1^	71 (84.5)
Migraine-like	40 (47.6)
Tension-type-like	11 (13.1)
Unclassifiable	20 (23.8)
Visual disturbances^1^	68 (81.0)
Abducens palsy^1^	13 (15.5)
Pulsatile tinnitus^1^	20 (23.8)
Ophthalmological findings	
Abnormal visual acuity^1^	18 (21.4)
Abnormal visual fields (perimetry)^1^	58 (69.0)
Papilledema^1^	73 (89.9)
Frisén-Scale^3^	3 (0–5)
Lumbar puncture opening pressure^3^	31 (28–39)

*BMI *Body mass index, *IIH *Idiopathic intracranial hypertension

^1^absolute number and percentage ^2^mean and standard deviation ^3^median and inter-quartile range

### Visual outcome

Overall, persistent visual impairment occurred in 58.3% and visual worsening in 13.1%.

In univariable analyses, patients with visual impairment at baseline (*n* = 62) had a significantly higher frequency of persistent visual impairment after 12 months (69.4% vs. 27.3%, p < 0.001) but not of further visual worsening (16.1% vs. 4.5%, *p* = 0.274). Neither age, sex, BMI, nor LP opening pressure were associated with persistent visual impairment or visual worsening.

Looking at the MRI signs, ONT was associated with a lower frequency of persistent visual impairment (30.6% vs. 68.6%, p < 0.001) as was the presence of ≥ 3 MRI features (74.2% vs. 48.7%, *p* = 0.049), while ES, POSD, PGF, TSS and ≥ 1 MRI feature were not (Supplemental Table 1). None of the MRI signs were associated with visual worsening.

In multivariable analyses, AICc suggested the models including baseline visual impairment as the most parsimonious ones concerning both persistent visual impairment and visual worsening, while age, BMI, and CSF opening pressure were not retained (Table 2). Here, baseline visual impairment was statistically significantly associated with an increased likelihood of persistent visual impairment (OR 6.24, 95% CI 2.09–18.7, p < 0.001). After adjusting for baseline visual impairment, neither any single MRI sign (ES, POSD, ONT, PGF, TSS) nor the number of detected MRI signs (≥ 1, ≥ 3) were significantly associated with either persistent visual impairment or visual worsening (Table 2).

As none of the five IIH-WOP patients showed persistent visual impairment or visual worsening, IIH-WOP could not be included into the regression models. Sensitivity analyses removing IIH-WOP patients did not significantly change results as was the case when removing patients requiring invasive treatment (*n* = 4), who all suffered persistent visual impairment and visual worsening after diagnosis.

**Table 2:** Multivariable regression models regarding visual outcome

	Persistent visual impairment^a^				Visual worsening^a^			
	Univariable		Multivariable		Univariable		Multivariable	
	OR (95% CI)^b^	*p*-value	OR (95% CI)^b^	*p*-value	OR (95% CI)^b^	*p*-value	OR (95% CI)^b^	*p*-value
**Age (per 5 years increase)**	0.99 (0.95–1.06)	0.992	not retained	n.a.	1.04 (0.97–1.08)	0.306	not retained	n.a.
**BMI (per point)**	0.99 (0.92–1.05)	0.699	not retained	n.a.	1.06 (0.98–1.17)	0.098	not retained	n.a.
**CSF opening pressure (per 5cmH2O)**	1.35 (0.92–1.89)	0.187	not retained	n.a.	1.44 (0.91–2.19)	0.492	not retained	n.a.
**Visual impairment at baseline**	**6.04 (2.04–17.8)**	**< 0.001**	**6.24 (2.09–18.7)**	**< 0.001**	4.04 (0.49–33.6)	0.196	3.46 (0.40–29.6)	0.258
**Empty sella**	0.73 (0.31–1.75)	0.481	0.61 (0.22–1.71)	0.347	2.51 (0.67–9.33)	0.170	1.84 (0.44–7.74)	0.406
**Optic nerve sheath distension**	0.43 (0.16–1.14)	0.089	0.63 (0.21–1.13)	0.089	0.36 (0.10–1.30)	0.119	0.36 (0.09–1.36)	0.131
**Optic nerve tortuosity**	**0.20 (0.08–0.52)**	**< 0.001**	0.50 (0.28–1.22)	0.094	0.39 (0.10–1.57)	0.183	0.39 (0.09–1.67)	0.205
**Posterior globe flattening**	0.72 (0.27–1.96)	0.524	0.57 (0.19–1.66)	0.300	1.15 (0.27–4.79)	0.852	1.19 (0.27–5.19)	0.818
**Transverse sinus stenosis**^**c**^	0.56 (0.21–1.48)	0.241	0.40 (0.13–1.21)	0.106	0.81 (0.20–3.33)	0.771	0.84 (0.19–3.72)	0.814
**≥ 1 MRI feature**	0.32 (0.10–1.08)	0.067	0.38 (0.15–1.18)	0.101	0.69 (0.16–2.92)	0.614	0.48 (0.10–2.23)	0.347
**≥ 3 MRI features**	**0.33 (0.12–0.92)**	**0.033**	0.32 (0.17–1.25)	0.163	1.39 (0.32–6.08)	0.663	1.04 (0.22–4.95)	0.961

*BMI *Body mass index, *CSF *Cerebrospinal fluid, *MRI *Magnetic resonance imaging, *N.a *not applicable, *OR *Odds ratio, *95% CI *95% confidence interval

^a^Calculated by Firth's bias-reduced multivariable binary logistic regression models with persistent visual impairment/ visual worsening as dependent variable and MRI features as independent variables (not present [reference category] vs. present). Corrected Akaike information criterion (AICc) used to select the most parsimonious models, i.e. which variables were retained.  ^b^Values above/below 1 indicate higher/lower probability of persistent visual impairment/ visual worsening. ^c^only available for 70 patients

### Headache outcome

Headache improvement was achieved in 83.3% and freedom of headache in 26.2%. Lower age was significantly correlated with headache improvement (rho − 0.256, *p* = 0.019) but not with freedom of headache. Patients with chronic headache at baseline (*n* = 43) had significantly lower frequency of headache freedom (14.6% vs. 37.2%, *p* = 0.025) but not headache improvement (81.4% vs. 85.4%, *p* = 0.772). Neither sex and BMI, nor LP opening pressure were associated with headache improvement and freedom. Also, neither headache frequency nor headache severity at baseline were significantly correlated with headache improvement. Of the five patients with IIH-WOP, all reached headache improvement but none freedom of headache.

Univariable analyses did not show any association between any MRI parameter (ES, POSD, ONT, PGF, TSS, ≥ 1 feature, ≥ 3 features) and either headache improvement or freedom of headache (Supplemental Table 2).

In multivariable analyses concerning headache improvement, AICc suggested a model including age and chronic headache at baseline as the most parsimonious model, while BMI, CSF opening pressure and baseline headache severity were not retained (Table 3). This model did not indicate any significant association between MRI features of IIH and headache improvement. Regarding freedom of headache, a model including chronic headache at baseline was the most parsimonious one, while age, BMI, CSF opening pressure and baseline headache severity were not retained (Table 3). Here, baseline chronic headache was significantly associated with a decreased likelihood of headache freedom (OR 0.48, 95% CI 0.17–0.91, *p* = 0.013), but there was no statistically significant association between any single MRI feature or their number and freedom of headache. As all five IIH-WOP patients showed headache improvement but none freedom of headache, IIH-WOP could not be included into the regression models. Sensitivity analyses removing IIH-WOP patients, patients with previous headache history, patients requiring invasive treatment as well as separately removing each headache phenotype group (migraine-like, tension-type-like, unclassifiable from the regression models did not significantly change the overall results or impact of single variables.

**Table 3:** Multivariable regression models regarding headache outcome

	Headache improvement^a^				Freedom of headache^a^			
	Univariable		Multivariable		Univariable		Multivariable	
	OR (95% CI)^b^	*p*-value	OR (95% CI)^b^	*p*-value	OR (95% CI)^b^	*p*-value	OR (95% CI)^b^	*p*-value
**Age (per 5 years increase)**	**0.95 (0.91–0.99)**	**0.047**	0.96 (0.91–1.01)	0.135	0.99 (0.95–1.04)	0.708	not retained	n.a.
**BMI (per point)**	0.98 (0.92–1.05)	0.619	not retained	n.a.	1.00 (0.94–1.06)	0.991	not retained	n.a.
**CSF opening pressure (per 5cmH2O)**	1.05 (0.84–1.33)	0.653	not retained	n.a.	1.15 (0.94–1.41)	0.175	not retained	n.a.
**Chronic headache at baseline**	1.34 (0.95–2.21)	0.105	1.39 (0.91–2.35)	0.197	**0.43 (0.13–0.92)**	**0.021**	**0.48 (0.17–0.91)**	**0.013**
**Baseline headache severity**	1.15 (0.68–1.95)	0.588	not retained	n.a.	0.69 (0.36–1.32)	0.258	not retained	n.a.
**Empty sella**	0.37 (0.11–1.22)	0.103	0.53 (0.15–1.91)	0.328	0.38 (0.13–1.09)	0.070	0.34 (0.11–1.09)	0.070
**Optic nerve sheath distension**	1.14 (0.34–3.78)	0.836	1.11 (0.32–3.88)	0.868	1.10 (0.39–3.11)	0.861	1.11 (0.39–3.14)	0.849
**Optic nerve tortuosity**	1.70 (0.52–5.59)	0.382	1.85 (0.54–6.29)	0.326	0.74 (0.28–1.98)	0.546	0.75 (0.28–2.01)	0.564
**Posterior globe flattening**	5.20 (0.64–42.4)	0.124	5.14 (0.62–42.5)	0.129	1.18 (0.39–3.54)	0.775	1.18 (0.39–3.56)	0.770
**Transverse sinus stenosis**^**c**^	2.47 (0.70–8.75)	0.162	2.55 (0.69–9.45)	0.160	0.42 (0.15–1.21)	0.109	0.43 (0.16–1.22)	0.111
**≥ 1 MRI feature**	1.00 (0.25–4.05)	0.999	1.35 (0.31–5.83)	0.687	0.90 (0.28–2.91)	0.863	0.94 (0.29–3.09)	0.918
**≥ 3 MRI features**	0.71 (0.19–2.62)	0.606	0.94 (0.24–3.69)	0.925	0.42 (0.15–1.21)	0.109	0.38 (0.13–1.15)	0.086

*BMI *Body mass index, *CSF *Cerebrospinal fluid, *MRI *Magnetic resonance imaging, *N.a *not applicable, *OR *Odds ratio, *VAS *Visual analogue scale, *95% CI *95% confidence interval. ^a^Calculated by Firth's bias-reduced multivariable binary logistic regression models with headache improvement/ freedom of headache as dependent variable and MRI features as independent variables (not present [reference category] vs. present). Corrected Akaike information criterion (AICc) used to select the most parsimonious models, i.e. which variables were retained. ^b^Values above/below 1 indicate higher/lower probability of headache improvement/ freedom of headache. ^c^only available for 70 patients

## Discussion

The aim of the present study was to determine whether MRI features of IIH are of value for predicting visual and headache outcome in a large and well-characterized real-world cohort.

To date, studies investigating prognostic value of MRI features of IIH largely focused on visual outcome and delivered conflicting results. One cross-sectional study reported that the presence of more than three MRI features correlated statistically significantly with the severity of vision loss at diagnosis, and a retrospective study indicated that TSS was associated with poor visual outcome [11, 12]. On the other hand, three retrospective studies found no association between MRI features and visual outcome [9, 10, 13]. These studies are limited by small sample sizes, a high likelihood of selection bias and lack adjustment for relevant confounders [9–13].

Applying thorough multivariable analyses based on a pre-defined approach designed to adjust for relevant confounders and multiple testing while also avoiding over-fitting, there was no association of any single MRI feature of IIH or their number with visual or headache outcome in the present study.

When analyzed in isolation, ONT and POSD displayed association with persistent visual impairment of our cohort. POSD and ONT, which is believed to occur due to pressure induced kinking by fixation of the optic nerve at proximal and distal points, presumably reflect increased CSF pressure in the optic nerve sheath [6]. While this would principally make POSD/ONT intriguing candidates for predicting visual outcome, multivariable analyses showed that this association is mostly mediated by visual impairment at baseline and, unfortunately, POSD/ONT did not provide independent additional prognostic value.

Overall, visual outcome in our cohort is well within the range of existing literature with about 60% displaying at least some degree of persistent visual impairment one year after diagnosis and a little over 10% suffering further visual worsening [10, 24, 25]. If visual impairment was already present at diagnosis, the odds of persistent visual impairment increased more than 6-fold constituting the only significant predictor of visual outcome in our cohort. This was expected and is in line with previous studies [10, 24–26].

Turning to headache outcome, improvement of headache frequency and/or severity was achieved in more than 80% of patients after one year of treatment in our cohort, while some degree of headache persisted in about three quarters of patients, both well in line with existing literature [27–30]. Studies investigating predictors of headache outcome in IIH are generally very scarce as most focus on visual outcome, likely because visual impairment is often considered more relevant and is also easier to measure in clinical practice. However, headache is indeed the main factor affecting quality of life in patients with IIH [31, 32]. Thus, identifying predictors of headache outcome is an unmet need in IIH. In our cohort, presence of headache already fulfilling criteria of chronic headache at baseline was the only factor remaining statistically significantly associated with headache outcome after conducting thorough multivariable analyses. Somewhat expectedly, chronic headache halved the odds for freedom of headache but not for headache improvement, as it is obviously easier to achieve a 50% reduction of monthly headache days or headache severity as opposed to less than one when starting with fifteen or more. This is in line with an earlier study, where longstanding headache was also associated with persistent headache after CSF shunting [33]. In agreement with previous findings, age, BMI and CSF opening pressure did not predict headache outcome [28]. Disappointingly, the investigated MRI features of IIH did not provide any independent prognostic information regarding headache improvement or freedom. It is well known that headache in IIH is not sufficiently explained by raised ICP alone [29, 30, 34]. As the pathophysiologic process underlying the development of MRI signs of IIH is likely primarily a correlate of raised ICP, this may explain the lack of an association with headache outcome.

### Strengths and limitations

The strengths of the VIIH database are the large sample size of a population-based cohort encompassing most IIH patients from our geographic area with close-meshed, standardized follow-up reflecting the whole spectrum of a real-world cohort [14, 35].

However, some limitations are be acknowledged. The retrospective analyses of data collected in clinical routine creates a variety of possible biases, e.g. diagnostic accuracy may be lower in this type of IIH cohort compared to a strictly prospective cohort, although these are mitigated by the standardized data collection and thorough quality control applied within the VIIH. Treatment regimens followed best practice recommendations but naturally varied inter-individually, potentially inducing bias. We could not adjust multivariable models for treatment parameters as this would have caused over-fitting. While sensitivity analyses did not indicate bias by invasive treatment (VP shunt), a confounding influence of weight loss and/or pharmacological treatment cannot be excluded. However, Rosenbaum bounds did indicate only a small potential impact of hidden bias not accounted for in the multivariable models. Thus, it is unlikely that a true prognostic effect of MRI signs was missed due to unaccounted confounding. Importantly, MRI scans were done in a real-world setting, which includes different scanners and field strengths (1.5 and 3T) and varying image acquisition protocols. Mitigating this potential cause of bias is the blinded rating by a senior neuroradiologist with extensive experience in IIH imaging. IIH-WOP remains a controversial diagnosis potentially representing a distinct phenotype. Since sample size (*n* = 5) in our cohort was insufficient to conduct subgroup analyses, we could only perform sensitivity analyses removing IIH-WOP patients to exclude a confounding effect on our results.

## Conclusion

MRI features of IIH are neither prognostic of visual nor headache outcome. Identifying reliable clinical or paraclinical predictors of outcome remains a critical area of need in IIH.

## Supplementary Information

**Additional file 1: Supplemental Table 1.** Association of MRI features of IIH with visual outcome.

**Additional file 2: Supplemental Table 2.** Association of MRI features of IIH with headache outcome.

## Data Availability

Data supporting the findings of this study are available from the corresponding author upon reasonable request by a qualified researcher and upon approval by data-clearing unit of the Medical University Vienna.
