# Supplementary material for: MRI features of idiopathic intracranial hypertension are not prognostic of visual and headache outcome
Source: J Headache Pain. 2023 Jul 28;24(1):97. doi: 10.1186/s10194-023-01641-x (PMC10386656; doi:10.1186/s10194-023-01641-x)
Supplement: Supplementary file 1 — Additional file 1: Supplemental Table 1. Association of MRI features of IIH with visual outcome. [file 10194_2023_1641_MOESM1_ESM.docx]

**Supplemental Table 1. Association of MRI features of IIH with visual outcome.**

|  | **Persistent visual impairment** | | | **Visual worsening** | | |
| --- | --- | --- | --- | --- | --- | --- |
|  | No (n=35) | Yes (n=49) | p-value^1^ | No (n=73) | Yes (n=11) | p-value^1^ |
| Empty sella | 48.6% | 40.8% | 0.511 | 41.1% | 63.6% | 0.201 |
| Perioptic subarachnoidal space distension | 77.1% | 59.2% | 0.104 | 69.9% | 45.5% | 0.168 |
| Optic nerve tortuosity | **68.6%** | **30.6%** | **0.001** | 49.3% | 27.3% | 0.209 |
| Posterior globe flattening | 28.6% | 22.4% | 0.612 | 24.7% | 27.3% | 0.999 |
| Transverse sinus stenosis (n=70) | 67.7% | 53.8% | 0.327 | 60.7% | 55.6% | 0.999 |
| ≥1 MRI feature | 88.6% | 71.4% | 0.066 | 79.5% | 72.7% | 0.695 |
| ≥3 MRI features | **74.2%** | **48.7%** | **0.049** | 59.0% | 66.7% | 0.732 |

^1^calculated by chi-square test. IIH: idiopathic intracranial hypertension. MRI: magnetic resonance imaging.
