# Supplementary material for: MRI features of idiopathic intracranial hypertension are not prognostic of visual and headache outcome
Source: J Headache Pain. 2023 Jul 28;24(1):97. doi: 10.1186/s10194-023-01641-x (PMC10386656; doi:10.1186/s10194-023-01641-x)
Supplement: Supplementary file 2 — Additional file 2: Supplemental Table 2. Association of MRI features of IIH with headache outcome. [file 10194_2023_1641_MOESM2_ESM.docx]

**Supplemental Table 2. Association of MRI features of IIH with headache outcome.**

|  | **Headache improvement** | | | **Freedom of headache** | | |
| --- | --- | --- | --- | --- | --- | --- |
|  | No (n=14) | Yes (n=70) | p-value^1^ | No (n=62) | Yes (n=22) | p-value^1^ |
| Empty sella | 64.3% | 40.0% | 0.140 | 50.0% | 27.3% | 0.082 |
| Perioptic subarachnoidal space distension | 64.3% | 67.1% | 0.999 | 66.1% | 68.2% | 0.999 |
| Optic nerve tortuosity | 35.7% | 48.6% | 0.558 | 48.8% | 40.9% | 0.623 |
| Posterior globe flattening | 7.1% | 28.6% | 0.173 | 24.2% | 27.3% | 0.780 |
| Transverse sinus stenosis | 41.7% | 63.8% | 0.201 | 66.0% | 45.0% | 0.116 |
| ≥1 MRI feature | 78.6% | 78.6% | 0.999 | 79.0% | 77.3% | 0.999 |
| ≥3 MRI features | 66.7% | 58.6% | 0.751 | 66.0% | 45.0% | 0.116 |

^1^calculated by chi-square test. IIH: idiopathic intracranial hypertension. MRI: magnetic resonance imaging.
